# The Impact of Temperature on Extracting Information From Clinical Trial Publications Using Large Language Models

**DOI:** 10.7759/cureus.75748

**Published:** 2024-12-15

**Authors:** Paul Windisch, Fabio Dennstädt, Carole Koechli, Christina Schröder, Daniel M Aebersold, Robert Förster, Daniel R Zwahlen

**Affiliations:** 1 Department of Radiation Oncology, Cantonal Hospital Winterthur, Winterthur, CHE; 2 Department of Radiation Oncology, Bern University Hospital, University of Bern, Bern, CHE

**Keywords:** large language models, natural language processing, temperature, text mining, transformer

## Abstract

Introduction

The application of natural language processing (NLP) for extracting data from biomedical research has gained momentum with the advent of large language models (LLMs). However, the effect of different LLM parameters, such as temperature settings, on biomedical text mining remains underexplored and a consensus on what settings can be considered “safe” is missing. This study evaluates the impact of temperature settings on LLM performance for a named entity recognition and a classification task in clinical trial publications.

Methods

Two datasets were analyzed using GPT-4o and GPT-4o-mini models at nine different temperature settings (0.00-2.00). The models were used to extract the number of randomized participants and classify abstracts as randomized controlled trials (RCTs) and/or as oncology-related. Different performance metrics were calculated for each temperature setting and task.

Results

Both models provided correctly formatted predictions for more than 98.7% of abstracts across temperatures from 0.00 to 1.50. While the number of correctly formatted predictions started to decrease afterward with the most notable drop between temperatures 1.75 and 2.00, the other performance metrics remained largely stable.

Conclusion

Temperature settings at or below 1.50 yielded consistent performance across text-mining tasks, with performance declines at higher settings. These findings are aligned with research on different temperature settings for other tasks, suggesting stable performance within a controlled temperature range across various NLP applications.

## Introduction

Using natural language processing (NLP) to extract data from biomedical research publications has garnered increasing interest, particularly with the advent of more powerful architectures, most notably large language models (LLMs) [1-3]. The task itself has been of interest for a long time as the ability to automatically extract and structure information, e.g., according to PICO (patient, intervention, control, outcome) characteristics, could improve various processes such as screening the literature for relevant publications, assessing adherence to reporting guidelines, and ultimately automating the process of evidence synthesis [4,5].

While the capability of LLMs for several of these text-mining tasks has been demonstrated previously, there is still relatively little information on the impact that different parameters or prompts might have [6,7]. A notable parameter of LLMs is the temperature of its softmax function that turns the "raw" outputs (i.e., the logits) into probabilities for the next token (i.e., the likeliest next part of a word). A high temperature leads to a flat probability distribution for the prediction of the next token, which makes the model more likely to choose less conventional options [8]. This may increase the creativity of the output but also make the behavior less predictive while increasing the risk of incoherent output. With a low-temperature setting, the model will choose only the most likely next token thus leading to a more predictive, coherent output with limited creativity. Although anecdotal reports suggest decreasing performance for certain tasks at higher temperatures, some publications report consistent performance across a broad temperature range, such as in answering multiple-choice questions or predicting clinical outcomes like in-hospital mortality from electronic health records [9,10]. Thus, there is no consensus on what temperature range can be considered “safe,” i.e., which range is unlikely to result in decreasing performance.

The purpose of this project was to evaluate the impact of temperature settings on text-mining tasks for clinical trial publications, specifically to determine whether performance remains consistent across a wide range of temperatures, as demonstrated for other tasks, and to identify the threshold beyond which performance begins to decline [9,10]. As most text-mining tasks generally fall into the categories of either named-entity recognition or classification, we used two dedicated datasets for these tasks.

## Materials and methods

Two datasets that had been annotated as part of previous projects by the author group were used to create tasks for the evaluation of two LLMs, namely Generative Pretrained Transformer 4 Omni (GPT-4o, OpenAI, San Francisco, United States) and GPT-4o mini at nine different temperature settings (0.00, 0.25, 0.50, 0.75, 1.00, 1.25, 1.50, 1.75, 2.00) [11-14]. The respective versions that were used were the latest versions available, namely gpt-4o-2024-05-13 and gpt-4o-mini-2024-07-18.

The first task was to extract the number of people who underwent randomization from the abstract of a publication reporting on a randomized clinical trial (RCT). To this end, a random sample of 996 RCTs from seven major journals (*British Medical Journal, JAMA, JAMA Oncology, Journal of Clinical Oncology, Lancet, Lancet Oncology, New England Journal of Medicine*) published between 2010 and 2022 were labeled. The abstracts were retrieved as a txt file from PubMed and parsed using regular expressions (i.e., expressions that match certain patterns in text). For each trial, the number of randomized trial participants was retrieved by looking at the abstract, followed by the full publication if the number could not be determined with certainty from the abstract. Two physician annotators carried out the annotation independently, and conflicts were resolved by discussing the differences afterward. 

The LLMs were called via the application programming interface (API) with the aforementioned temperatures and max_tokens set to 10 to stop the LLM in case of hallucinations associated with producing large amounts of incoherent output. All other API parameters were left at their default, which can be found in the documentation [15]. The system prompt was the following: “You will be provided with the abstract of an RCT. Your task will be to extract the number of people who underwent randomization. If this number is not explicitly mentioned, you may use other numerical information (e.g. the number of total participants or adding up the number of patients in each arm). Please return only the number as a single integer. If no information is available, please return null."

The user prompt was the respective abstract. The raw responses were stored and afterward, each raw response was converted into an integer unless the conversion failed, e.g., due to the raw response being equal to “null” or due to non-numerical hallucinations.

The results were evaluated against the ground truth created by the human annotators. The percentage of correctly formatted, numerical predictions was calculated as well as performance metrics like the mean absolute percentage error (MAPE) and the proportion of predictions that fell within a certain percentage of the ground truth.

The second task was to classify an abstract regarding whether or not it was reported on an RCT and/or an oncology topic. To this end, a random sample of 900 publications from the aforementioned seven major journals published between 2010 and 2022 were annotated. Publications that described RCTs received the label “RCT.” Publications that covered oncological topics received the label “ONCOLOGY.” Trials that fulfilled both criteria were assigned both labels. Trials that were neither RCTs nor covered oncology topics were assigned no label. The two labels were chosen as each label poses different requirements to the LLM: For the oncology label, the model does not need a deep contextual understanding but can rather make a prediction based on the presence of certain words that are associated with oncology publications, such as “cancer” or words related to staging and antineoplastic therapies. In order to assign the RCT label, the model can not simply rely on the presence of words and phrases like “randomized” or “primary endpoint” as these might also be present in other articles such as meta-analyses of RCTs. 

Annotation was based on the title and abstract, which were also retrieved as a txt file from PubMed and parsed using regular expressions. Due to the relatively simple annotation process, annotation was carried out by a single physician annotator. The API call to the LLMs used the same settings as those for the first task. The user prompt was again the abstract. The system prompt was the following: "You will be provided with the abstract of a medical publication. Your task will be to determine if the abstract reports on a RCT. If the abstract reports on a systematic review or meta-analysis of RCTs or a commentary/editorial, return false. In addition, you will be asked to determine if the abstract focuses on an oncology topic which includes all papers dealing with the prevention, diagnosis or treatment of solid or hematologic cancers. Your response should be a list of two boolean values (True or False), the first indicating if the paper is an RCT and the second indicating if the paper is oncology-related. The list should be enclosed in brackets and separated by a comma, e.g. [True, False]."

The raw responses were stored and afterward, the two boolean values were extracted unless the extraction failed due to incorrect formatting. The results were evaluated against the ground truth by computing the proportion of correctly formatted predictions (i.e., a single integer or a list of two booleans in brackets separated by a comma) as well as the confusion matrices for each label and several performance metrics (accuracy, precision, recall, and F1 score).

All programming was performed in Python (version 3.11.5) using, among others, the pandas (version 2.1.0) and openai (version 1.40.3) packages. This article was previously posted to the medRxiv preprint server on October 23, 2024.

## Results

**Figure 1:**
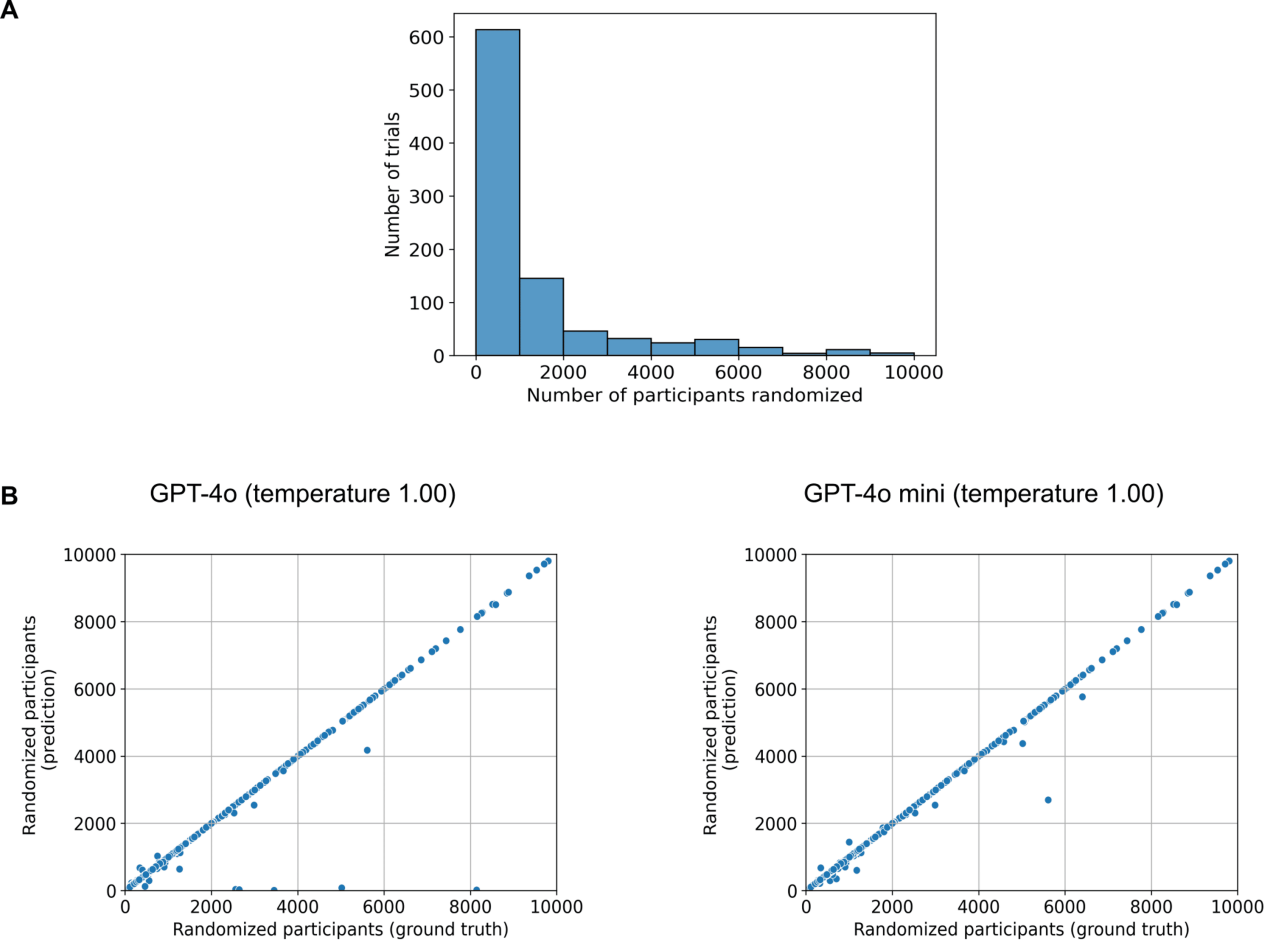
Predicting or extracting the number of randomized trial participants A) Histogram of the trials and the respective number of randomized participants. B) Scatterplots where each dot represents a trial with its x-coordinate representing how many people were randomized and the y-coordinate representing what GPT-4o (left) or GPT-4o mini (right) predicted in terms of how many people were randomized at temperature 1.00. To ensure better visualization of the range that most trials fall into, only trials and predictions with less than 10,000 randomized participants are displayed.

The median number of people who underwent randomization was 668 with an interquartile range (IQR of 300-1836) and a histogram of the respective number of people who underwent randomization in each trial is presented in Figure 1A. The percentage of trials with correctly formatted numerical predictions made by GPT-4o was almost constant between temperatures 0.00 and 1.50, ranging from 98.7% to 99.0%. The first noticeable drop occurred at temperature 1.75 with 95.6% with a further drop to 89.2% at temperature 2.00. The same pattern could be seen with GPT-4o mini where temperatures between 0.00 and 1.50 resulted in trials with correctly formatted numerical predictions between 99.0% and 99.1% and drops at 1.75 as well as 2.00 (97.5% and 90.2%, respectively). A scatterplot of the predictions of the LLMs compared to the ground truth is presented in Figure 1B. The complete performance metrics are presented in Table 1.

**Table 1:** Performance of GPT-4o and GPT-4o mini when asked to extract the number of patients who were randomized from the abstract of a publication reporting on an RCT “Predicted trials” indicates the percentage of trials for which a correctly formatted, numerical prediction was returned. RCT, randomized controlled trial; MAPE, mean absolute percentage error

Temperature	Predicted trials, %	MAPE, %	Predictions within 10% from ground truth, %	Predictions within 1% from ground truth, %
GPT-4o				
0.00	98.7	1.7	96.9	94.1
0.25	98.8	1.8	97.0	94.0
0.50	98.7	1.5	97.3	94.3
0.75	99.0	2.3	96.5	93.6
1.00	98.8	2.0	96.5	93.7
1.25	98.7	1.7	96.6	93.7
1.50	98.7	3.8	96.2	93.4
1.75	95.6	1.8	96.7	94.2
2.00	89.2	1.0	98.0	95.3
GPT-4o mini				
0.00	99.1	1.5	96.8	92.7
0.25	99.1	1.5	96.8	92.7
0.50	99.0	1.3	97.0	93.1
0.75	99.1	1.4	96.9	92.7
1.00	99.1	1.4	96.7	92.9
1.25	99.1	1.3	97.0	93.1
1.50	99.1	1.5	96.7	92.8
1.75	97.5	1.6	96.4	92.4
2.00	90.2	1.6	96.7	92.9

When analyzing only the correctly formatted predictions, the performance in terms of MAPE and the proportion of predictions within a certain margin of error did not show a major drop beyond a certain temperature. On the contrary, for GPT-4o, a temperature of 2.00 resulted in the lowest MAPE and the highest proportion of predictions within 10% and 1% of the ground truth.

**Figure 2:**
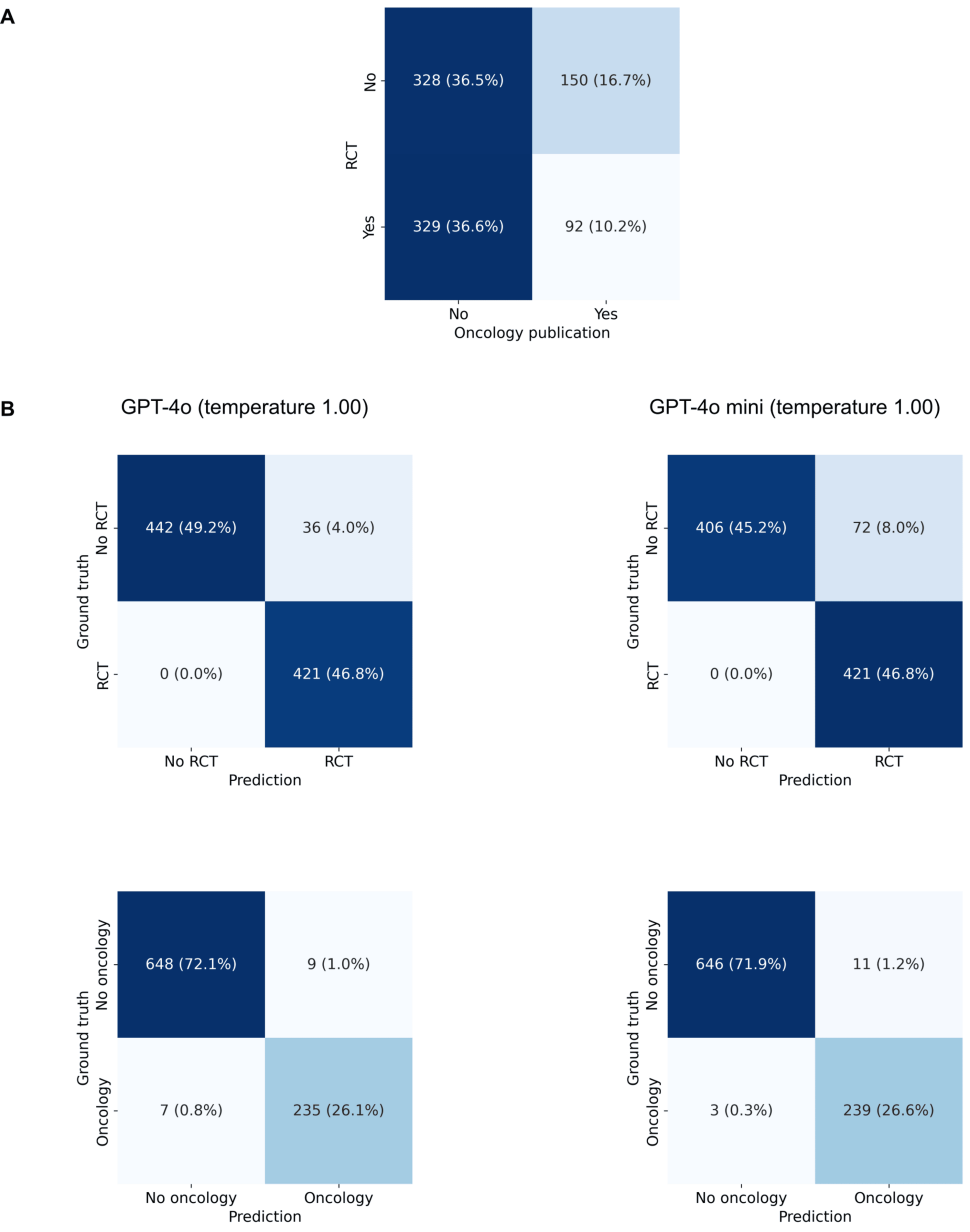
Trial classification Confusion matrix regarding whether or not the abstract of a publication reports on an RCT and whether or not it reports on an oncology topic. B) Confusion matrices for the predictions of GPT-4o (left) or GPT-4o mini (right) at a temperature of 1.00 regarding whether or not the abstract of a publication reports on an RCT (top) and whether or not it reports on an oncology topic (bottom). RCT, randomized controlled trial

A confusion matrix on the distribution of RCTs and oncology trials is presented in Figure 2A. 46.8% of trials were RCTs and 26.9% covered an oncology topic. The predictions of the LLMs compared to the ground truth for each label are presented in the confusion matrices in Figure 2B. The performance metrics are presented in Table 2 (RCT) and Table 3 (oncology). The classification task resulted in trials with correctly formatted predictions in 100% of abstracts with both models and labels for temperatures at or below 1.25. The biggest drop occurred again between temperatures 1.75 (98.9% for GPT-4o and 97.7% for GPT-4o mini) and 2.00 (93.3% for GPT-4o and 92.4% for GPT-4o mini) for both labels. The F1 scores for the label RCT ranged from 0.956 to 0.960 for GPT-4o and 0.914 to 0.921 for GPT-4o mini. The F1 scores for the label oncology ranged from 0.965 to 0.977 for GPT-4o and 0.964 to 0.972 for GPT-4o mini.

**Table 2:** Performance of GPT-4o and GPT-4o mini when asked to predict if an abstract of a publication reported on an RCT “Predicted trials” indicates the percentage of trials for which a correctly formatted prediction was returned. RCT, randomized controlled trial

Temperature	Predicted trials, %	Accuracy	Precision	Recall	F1 score
GPT-4o					
0.00	100.0	0.957	0.915	1.000	0.956
0.25	100.0	0.959	0.919	1.000	0.958
0.50	100.0	0.957	0.915	1.000	0.956
0.75	100.0	0.958	0.917	1.000	0.957
1.00	100.0	0.960	0.921	1.000	0.959
1.25	100.0	0.960	0.921	1.000	0.959
1.50	99.9	0.961	0.923	1.000	0.960
1.75	98.9	0.960	0.921	1.000	0.959
2.00	93.3	0.957	0.916	1.000	0.956
GPT-4o mini					
0.00	100.0	0.915	0.847	1.000	0.917
0.25	100.0	0.918	0.851	1.000	0.919
0.50	100.0	0.915	0.847	1.000	0.917
0.75	100.0	0.919	0.852	1.000	0.920
1.00	100.0	0.920	0.854	1.000	0.921
1.25	100.0	0.917	0.849	1.000	0.918
1.50	99.6	0.918	0.852	1.000	0.920
1.75	97.7	0.916	0.849	1.000	0.918
2.00	92.4	0.911	0.842	1.000	0.914

**Table 3:** Performance of GPT-4o and GPT-4o mini when asked to predict if an abstract of a publication reported on an oncology topic “Predicted trials” indicates the percentage of trials for which a correctly formatted prediction was returned.

Temperature	Predicted trials, %	Accuracy	Precision	Recall	F1 score
GPT-4o					
0.00	100.0	0.983	0.967	0.971	0.969
0.25	100.0	0.982	0.963	0.971	0.967
0.50	100.0	0.984	0.967	0.975	0.971
0.75	100.0	0.983	0.963	0.975	0.969
1.00	100.0	0.982	0.963	0.971	0.967
1.25	100.0	0.983	0.963	0.975	0.969
1.50	99.9	0.981	0.963	0.967	0.965
1.75	98.9	0.988	0.967	0.988	0.977
2.00	93.3	0.985	0.970	0.974	0.972
GPT-4o mini					
0.00	100.0	0.983	0.952	0.988	0.970
0.25	100.0	0.983	0.952	0.988	0.970
0.50	100.0	0.984	0.956	0.988	0.972
0.75	100.0	0.982	0.945	0.992	0.968
1.00	100.0	0.984	0.956	0.988	0.972
1.25	100.0	0.984	0.956	0.988	0.972
1.50	99.6	0.980	0.937	0.992	0.964
1.75	97.7	0.981	0.940	0.992	0.965
2.00	92.4	0.983	0.953	0.987	0.970

## Discussion

In this study, temperatures at or below 1.50 yielded comparable results and the most pronounced drop in performance occurred between temperatures 1.75 and 2.00. Notably, the drop in performance only occurred with regard to the proportion of correctly formatted predictions while the error metrics of the correctly formatted predictions remained constant and in some cases even improved. A possible explanation for this seemingly counterintuitive improvement could be that abstracts in which the number of people randomized is not explicitly stated, requiring the model to infer it from the available information, are more likely to trigger hallucinations. Thus, more difficult abstracts end up with incorrectly formatted predictions, which results in better performance when looking only at the correctly formatted predictions. These findings regarding text mining are largely consistent with Patel and colleagues who saw a consistent performance of various LLMs (GPT-4, GPT-3.5, and Llama-3-70b) for various clinical tasks across a temperature range from 0.2 to 1.0 as well as Renze and colleagues who saw consistent results for an even wider array of LLMs between temperatures of 0.0 to 1.0 [9,10]. If a hallucination occurred, it consisted of nonsensical output that sometimes also contained the desired prediction, usually at the beginning (e.g. “849 Duits.BOTTOM XTrella Brent涩 уру instituto.zaxxer” or ”[True, False]ynamicsCookingocious_student yhtä”).

A second insight of this observation is that specifying a desired output format via the prompt could facilitate the detection of hallucinations. This raises the question of whether forcing the LLM to adhere to a specific output format, such as using features like Structured Outputs or JSON mode, could eliminate this behavior and result in more incorrect predictions being correctly formatted. This topic warrants further research. 

Another interesting observation is the difference in performance between GPT-4o and GPT-4o-mini when predicting whether an abstract reports on an RCT, whereas their performance was similar when predicting whether the abstract pertains to an oncology topic. A possible explanation for this is that determining if an abstract pertains to an oncology topic is a simpler task, as it mainly requires recognizing which words in the abstract are associated with oncology. To determine if an abstract reports on an RCT, one cannot only rely on the terminology but has to understand the context. As an example, the phrase “randomized controlled trial” can occur in an abstract reporting on an RCT, but also in a systematic review that includes RCTs. Therefore, it seems plausible that the more powerful model, i.e., GPT-4o, performs better at this task.

This study is limited by the fact that only OpenAI models were used to analyze the different temperature settings. However, the evaluation studies of different temperature settings for other tasks such as those mentioned previously indicate that the findings are likely to generalize to other architectures [9,10]. Furthermore, other factors influencing the output of the LLM, such as the choice of model, additional model parameters, or different prompts, were not investigated [16]. The specific setting of these factors can influence each other as well as the output and, in turn, the performance and should be investigated in the future. While it seems unlikely that changes in these factors would yield substantially different results, one should be aware of this fact when interpreting this study and likely do at least a focused evaluation of different parameters for the task that one is trying to accomplish.

As a potential outlook, one could try to create more robust text-mining workflows by sending the same or slightly varied prompts to models with different temperature settings in the seemingly safe range of temperatures from 0.00 to 1.50. If all models are in agreement, the prediction is considered correct. If there is a disagreement, a manual review is triggered. This workflow could also be implemented to reduce costs if, e.g., three predictions from small, cheaper models at different temperatures are requested and a more expensive model is only used if there is a disagreement.

## Conclusions

In conclusion, temperature settings at or below 1.50 seem to result in comparable performance for extracting information from medical publications. These findings are aligned with research on different temperature settings for other tasks, suggesting that a safe temperature range may be consistent across a variety of applications. A focused evaluation of different parameters for the task that one is trying to accomplish when using an LLM is recommended considering the uncertainty regarding the relationship between different parameters.
